# Experience with crossmodal statistics reduces the sensitivity for audio-visual temporal asynchrony

**DOI:** 10.1038/s41598-017-01252-y

**Published:** 2017-05-03

**Authors:** Boukje Habets, Patrick Bruns, Brigitte Röder

**Affiliations:** 1 Biological Psychology and Neuropsychology, Faculty for Psychology and Human Movement Science, University of Hamburg, Von-Melle-Park 11, 20146 Hamburg, Germany; 2 Biological Psychology & Cognitive Neuroscience, Faculty for Psychology and Sport Science, University of Bielefeld, Universitätsstraße 25, 33615 Bielefeld, Germany; 3 Department of Cognitive, Linguistic & Psychological Sciences, Brown University, 190 Thayer Street, Providence, RI 02912 USA

## Abstract

Bayesian models propose that multisensory integration depends on both sensory evidence (the likelihood) and priors indicating whether or not two inputs belong to the same event. The present study manipulated the prior for dynamic auditory and visual stimuli to co-occur and tested the predicted enhancement of multisensory binding as assessed with a simultaneity judgment task. In an initial learning phase participants were exposed to a subset of auditory-visual combinations. In the test phase the previously encountered audio-visual stimuli were presented together with new combinations of the auditory and visual stimuli from the learning phase, audio-visual stimuli containing one learned and one new sensory component, and audio-visual stimuli containing completely new auditory and visual material. Auditory-visual asynchrony was manipulated. A higher proportion of simultaneity judgements was observed for the learned cross-modal combinations than for new combinations of the same auditory and visual elements, as well as for all other conditions. This result suggests that prior exposure to certain auditory-visual combinations changed the expectation (i.e., the prior) that their elements belonged to the same event. As a result, multisensory binding became more likely despite unchanged sensory evidence of the auditory and visual elements.

## Introduction

Most of our percepts of the world are multisensory. For example, preparing a meal provides us with tactile, visual, olfactory and auditory information (e.g., when washing and cutting vegetables). Receiving information from different senses is highly advantageous since events become more salient which in turn enables quicker and more adequate responses to crossmodal compared to unimodal stimuli^1, 2^. Despite a huge body of research (e.g. ref. 3) the question of how the brain combines information from the different senses into a coherent percept is still not fully understood. It has been suggested that so-called supramodal features, that is, features which are redundantly coded by multiple sensory systems, are used to judge whether or not inputs from different modalities originate from the same source. Examples of supramodal features are time, space, number and meaning^4^: the higher the overlap of inputs from different sensory modalities with regard to supramodal features, the more likely the brain will treat these inputs as coming from the same source. For example, a bouncing ball makes a sound each time the ball hits the ground and both sound and visual motion appear at the same location. Hence it is likely to perceive one object producing both types of sensory input.

In addition to supramodal features, previous experience with specific crossmodal combinations might influence multisensory binding: if certain crossmodal combinations repeatedly coincide in the environment, we learn through exposure that specific sensory events belong together. For example, we learn the combination of someone’s face and voice through numerous instances of exposure to this specific audio-visual combination^5^. Thus, besides the supramodal features mentioned above (time, space, number and meaning), prior knowledge, derived from experience with and exposure to the environment, might create an expectation (prior) about the co-occurrence of unimodal events^6^.

The influence of prior knowledge on multisensory binding has mostly been investigated by means of speech stimuli. This approach has been chosen because speech is a class of highly overlearned multisensory stimuli^7^. When comparing gender-matched and gender-mismatched audio-visual speech stimuli, it turned out that judging the temporal order of gender-incongruent speech stimuli was easier than judging the temporal order of gender-congruent speech stimuli^8^. Moreover, the stimulus onset asynchrony (SOA) between an auditory and visual stimulus needed to be larger for semantically congruent audio-visual speech pairs (approximately 200 ms) than for incongruent audio-visual speech pairs (approximately 160 ms) before participants noticed a difference in temporal onset^9, 10^. It was postulated that the higher complexity of speech stimuli makes them more robust against temporal asynchronies^7^. So far, similar effects have not been found for semantically meaningful non-speech stimuli^11–13^. For example, video sequences showing a hammer crushing a block of ice or a bouncing ball were combined with matching and non-matching sounds: the visual presentation of a bouncing ball was combined with the sound of the bouncing ball or with the sound of a hammer crushing a block of ice (and vice versa)^13^. No differences were found in temporal order judgments between such semantically congruent and incongruent crossmodal stimuli. These results suggest that learning-induced multisensory binding in language might be highly specific. Alternatively, however, it could be argued that object sounds are less familiar and/or their temporal dynamics less complex than audio-visual speech. In order to fully understand the influence of prior knowledge on multisensory binding, it is therefore necessary to experimentally manipulate crossmodal statistics, rather than to use overlearned stimuli such as speech or object stimuli. Indeed, experimentally associated arbitrary auditory and visual stimuli seem to result in early multisensory interactions^14^.

The present study tested the influence of prior association learning of arbitrary auditory and visual stimulus combinations on the ability to segregate these stimuli in time. Participants were exposed to artificial audio-visual combinations (videos) prior to a simultaneity judgment task. During this learning phase, participants were instructed to pay attention to the crossmodal *combination* of the auditory-visual stimuli (see Supplementary Table S1). During the subsequent test phase participants performed a simultaneity judgment task: they had to indicate whether or not the auditory and visual parts were synchronously presented. Nine SOAs in steps of 100 ms (−400 to +400 ms) were used. During the test phase the learned audio-visual stimuli (e.g., A(auditory)1 V(visual)1 and A2V2; numbers indicate exemplars and are used here to illustrate the different conditions) were presented amidst new combinations of the previously encountered auditory and visual stimuli (e.g., A1V2 and A2V1), as well as crossmodal stimuli containing one known and one new sensory component (e.g., A1V13, A13V1) (see Supplementary Table S1). Note that the auditory and visual elements of crossmodal stimuli were the same in the learned and the newly combined crossmodal stimuli; what differed was whether the specific crossmodal combination was learned or not. Crossmodal stimuli containing completely new auditory and visual stimuli (e.g., A7V7) were added as a baseline condition as well. After completion of the test phase, participants watched all crossmodal stimuli and indicated whether or not they had been exposed to them during the initial learning phase. Only participants who correctly recognized all audio-visual stimuli from the learning phase were included in the analysis. We expected a higher likelihood of perceived simultaneity for the learned crossmodal stimuli in comparison to any other condition due to a stronger prior^6, 15^ or assumption of unity^4^ that their elements belong to the same object.

## Results

### Reaction times

The mean reaction time (RT) of the simultaneity judgments was 1731 ms (*SD* = 287 ms). An ANOVA with factors First Modality (vision vs. audition presented first), SOA (100, 200, 300, 400 ms), and Condition (audio-visual learned (L), audio-visual newly combined (NC), audio-visual visual_learned (V-l), audio-visual auditory_learned (A-l) and audio-visual new (N)) did not reveal any significant differences in RTs (First Modality: *F*(1, 11) = 3.4, *p* = 0.09; SOA: *F*(3, 33) = 1.2, *p* = 0.32; Condition: *F*(4, 44) = 0.5, *p* = 0.53) and no significant interactions (all *p*s > 0.12). This result suggests that the simultaneity judgment results reported in the next section are not due to a speed accuracy trade-off or a lack of compliance with the task instructions.

### Perceived simultaneity judgments

**Figure 1:**
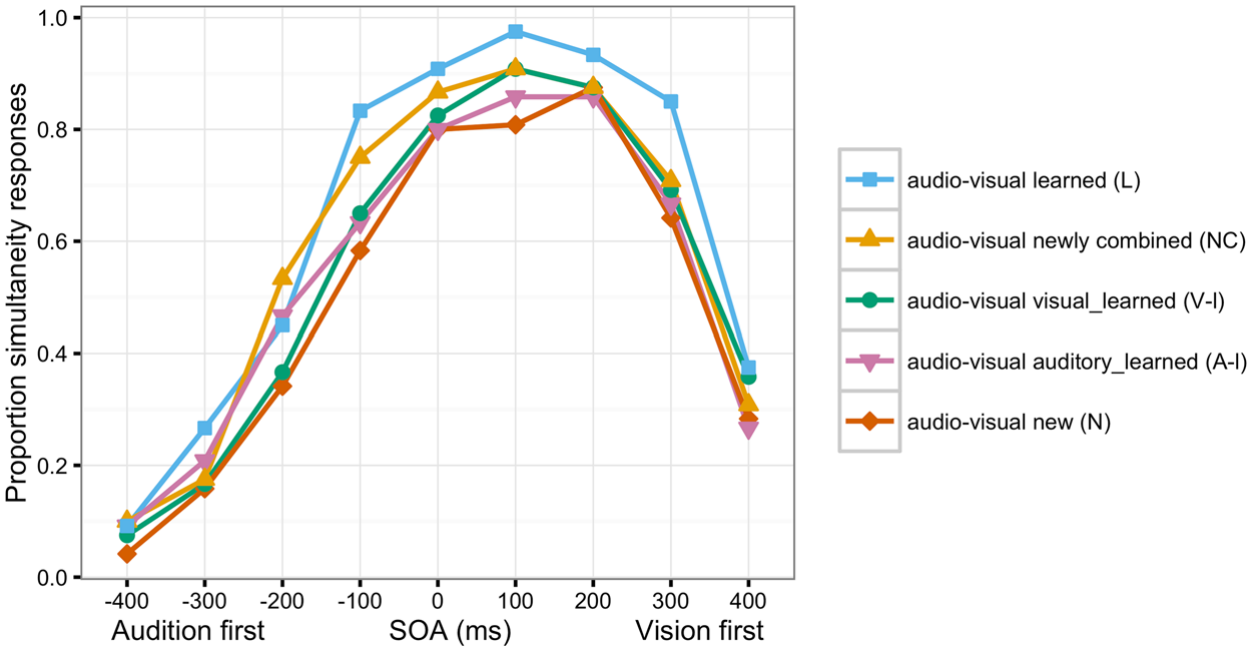
Mean proportion of ‘perceived simultaneity’ responses as a function of stimulus onset asynchrony (SOA) and first modality (negative values = auditory stimulus first, positive values = visual stimulus first) for audio-visual learned (L), audio-visual newly combined (NC), audio-visual visual_learned (V-l), audio-visual auditory_learned (A-l) and audio-visual new (N) stimuli.

An ANOVA with factors First Modality (vision vs. audition presented first), SOA (100, 200, 300, 400 ms), and Condition (audio-visual learned (L), audio-visual newly combined (NC), audio-visual visual_learned (V-l), audio-visual auditory_learned (A-l) and audio-visual new (N)) revealed significant main effects of First Modality (*F*(1, 11) = 148.1, *p* < 0.001), SOA (*F*(3, 33) = 162.4, *p* < 0.001) and Condition (*F*(4, 44) = 5.3, *p* = 0.02). Significant interactions between First Modality x SOA (*F*(3, 33) = 10.4, *p* = 0.002) and SOA x Condition (*F*(12, 132) = 2.6, *p* = 0.02) were found (see Fig. 1 and Supplementary Table S2). The main effect of First Modality was due to a higher likelihood to perceive simultaneity when visual preceded auditory information. To further explore the interactions with SOA, we conducted separate analyses for each SOA factor level and audition vs. vision leading (see Table 1). If vision preceded audition, learned videos were significantly more often judged as simultaneous than newly combined videos (*p* < 0.05 for SOAs 100 and 300 ms (Vision first)). Moreover, learned videos differed from all other videos (*p*s < 0.05 for SOAs −100 (Audition first) and 100 and 300 ms (Vision first); see Table 1). The newly combined videos were judged as simultaneous more often than the new videos for the SOAs −400, −300 and −100 ms (Audition first).

**Table 1:** Results of two-tailed t-tests for all conditions and SOAs.

Conditions		SOA							
		−400	−300	−200	−100	100	200	300	400
L – NC	*t*(11)	−0.18	1.42	−0.99	1.45	**2.35**	1.63	**4.53**	1.48
	*p*	0.862	0.183	0.344	0.175	0.039*	0.132	0.001*	0.166
	Cohen’s *d*	0.05	0.41	0.29	0.42	**0.68**	0.47	**1.31**	0.43
L – V_l	*t*(11)	0.33	1.41	1.13	**3.12**	2.15	1.40	**3.08**	0.16
	*p*	0.748	0.185	0.282	0.010*	0.054	0.189	0.011*	0.873
	Cohen’s *d*	0.10	0.41	0.33	**0.90**	0.62	0.40	**0.89**	0.05
L – A_l	*t*(11)	0.00	0.83	−0.16	**3.32**	**2.88**	2.02	**3.12**	1.46
	*p*	1	0.423	0.873	0.007*	0.015*	0.069	0.010*	0.173
	Cohen’s *d*	0.00	0.24	0.05	**0.96**	**0.83**	0.58	**0.90**	0.42
L – N	*t*(11)	1.39	1.50	1.46	**5.00**	**2.46**	0.98	**3.65**	1.61
	*p*	0.191	0.162	0.173	0.001*	0.032*	0.349	0.004*	0.136
	Cohen’s *d*	0.40	0.43	0.42	**1.44**	**0.71**	0.28	**1.05**	0.46
NC – V_l	*t*(11)	0.67	0.15	**3.86**	1.97	0.00	0.00	0.52	−0.53
	*p*	0.515	0.881	0.003*	0.074	1	1	0.615	0.606
	Cohen’s *d*	0.19	0.04	**1.11**	0.57	0.00	0.00	0.15	0.15
NC – A_l	*t*(11)	0.23	−1.00	1.88	2.18	1.07	0.56	0.70	0.73
	*p*	0.820	0.339	0.087	0.052	0.309	0.586	0.499	0.480
	Cohen’s *d*	0.07	0.29	0.54	0.63	0.31	0.16	0.20	0.21
NC – N	*t*(11)	**2.24**	0.39	**3.73**	**3.86**	1.59	0.00	1.34	0.54
	*p*	0.046*	0.701	0.003*	0.003*	0.139	1	0.207	0.600
	Cohen’s *d*	**0.65**	0.11	**1.08**	**1.11**	0.46	0.00	0.39	0.16
V_l – A_l	*t*(11)	−0.80	−0.67	−2.03	0.32	1.59	0.56	0.45	1.54
	*p*	0.438	0.516	0.067	0.755	0.139	0.586	0.660	0.152
	Cohen’s *d*	0.23	0.19	0.59	0.09	0.46	0.16	0.13	0.44
V_l – N	*t*(11)	1.30	0.32	0.61	1.15	1.77	0.00	1.39	0.99
	*p*	0.220	0.755	0.555	0.276	0.104	1	0.191	0.345
	Cohen’s *d*	0.38	0.09	0.18	0.33	0.51	0.00	0.40	0.28
A_l – N	*t*(11)	1.39	1.11	**2.45**	1.00	1.25	−0.39	0.67	−0.48
	*p*	0.191	0.293	0.032*	0.339	0.236	0.701	0.515	0.638
	Cohen’s *d*	0.40	0.32	**0.71**	0.29	0.36	0.11	0.19	0.14

Note. SOA = stimulus onset asynchrony (negative values = auditory stimulus first, positive values = visual stimulus first); L = audio-visual learned; NC = audio-visual newly combined; V-l = audio-visual visual_learned; A-l = audio-visual auditory_learned; N = audio-visual new.

*p < 0.05 (values of significant pairwise comparisons are in boldface).

### Temporal binding window

**Figure 2:**
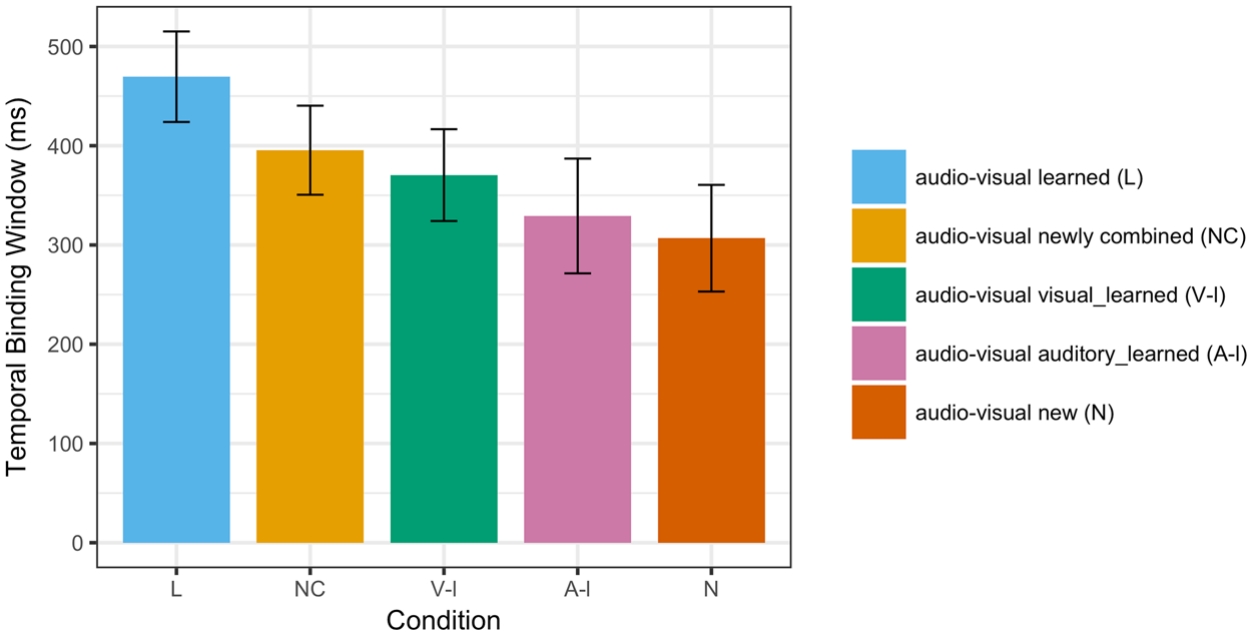
Mean size of the temporal binding window (TBW) for audio-visual learned (L), audio-visual newly combined (NC), audio-visual visual_learned (V-l), audio-visual auditory_learned (A-l) and audio-visual new (N) stimuli. Error bars denote standard errors of the mean.

In addition to the analysis of mean proportions of simultaneity judgments reported above, temporal binding windows (TBWs) were derived for each participant and condition as a singular measure of multisensory binding^16^. Two separate sigmoid functions were fitted to auditory-leading and visual-leading SOAs, and TBWs were calculated as the difference (in ms) between the right and the left curve at 75% perceived simultaneity (see Data Analysis for details). A one-way repeated-measures ANOVA revealed that TBWs significantly differed between conditions, *F*(4, 44) = 5.80, *p* = 0.015 (see Fig. 2). The TBW in the learned (L) condition was significantly larger than in any other condition (all *p*s < 0.05; Cohen’s *d*s ≥ 0.71). In addition, TBWs in the newly combined (NC) and the visual_learned (V-l) conditions were significantly larger than in the new (N) condition (both *p*s < 0.05; Cohen’s *d*s ≥ 0.67).

## Discussion

The aim of the present study was to test the hypothesis that the probability of multisensory binding of an auditory and a visual stimulus is changed (despite unchanged sensory evidence of the auditory and visual elements) if the prior for this specific audio-visual combination to co-occur is experimentally manipulated. During a learning phase, participants were instructed to pay attention to audio-visual combinations of six repeatedly presented audio-visual dynamic stimuli (videos). The test phase involved the learned videos as well as new combinations of the same auditory and visual stimuli, audio-visual stimuli with one previously encountered and one new component, and audio-visual combinations comprising both a new auditory and a new visual element. The key result of the present study was the higher probability to perceive audio-visual stimuli as simultaneous when the audio-visual combination had been encountered prior to the simultaneity judgment task compared to when the same auditory and visual elements occurred in non-learned combinations. The sole difference between the learned and the newly combined videos was the learning experience regarding specific stimulus combinations, whereas the sensory information, that is the auditory and visual elements, was identical in both conditions. Therefore, the difference in perceived synchrony between these two conditions must stem from an increase in the likelihood to bind previously encountered audio-visual combinations.

These results resemble findings with speech stimuli showing a lower temporal order judgment performance for congruent vs incongruent audio-visual speech combinations^8, 9, 11, 16^. However, most of the previous studies^12^ did not show any effects of semantic knowledge on binding of multisensory object actions: For example, no differences in cross-modal temporal discrimination were found when making temporal order judgments about matching (e.g., visual stimulus of a bouncing ball with the semantically correct sound) versus non-matching (same visual stimulus with a semantically incorrect sound) object actions^12^. However, the ability to recognize auditory and visual components of cross-modal stimuli to the same degree and with the same ease is hard to control in studies involving meaningful natural stimuli. For example, while auditory speech perception is superior to lipreading, visual object recognition generally outperforms auditory object recognition^17^. In contrast to these studies, the present experiment used artificial stimuli and arbitrary combinations of auditory and visual stimuli. By holding sensory stimulation constant and by experimentally manipulating association strength, we were able to provide evidence that an experimentally induced a priori expectation of an auditory and visual input to co-occur increased the likelihood for multisensory binding as assessed with a simultaneity judgement task: In other words, the stimulus onset asynchrony between auditory and visual onset had to be larger for learned audio-visual combinations in comparison to newly combined audio-visual pairs for participants to notice a difference in temporal onset and, thus, to segregate these events in time. Hence, association learning altered the “assumption of unity”^4^. The effects of prior experience (association learning) can be considered in the framework of causal inference models^6, 15^. In the context of multisensory perception, these models deal with the question of how the brain assigns multiple sensory inputs to one or multiple sources. If, for example, auditory and visual input originate from the same event they should be assigned to a common source and thus should be integrated. In contrast, if auditory and visual input originate from distinct events, they should be assigned to distinct sources and should, thus, not be integrated. Causal inference models, as typical Bayesian models, allow for a prior defining the probability of a common cause. Since we kept the sensory evidence (likelihood) of the auditory and visual elements constant, the increase in the proportion of simultaneity judgements, and thus the change in multisensory binding, must be attributed to an increase in the prior for a common cause. Our results are furthermore reminiscent of the observation that typical multisensory illusions, such as the ventriloquist effect, are larger if participants report auditory and visual stimuli occurring from the same event^18^. However, these studies manipulated the sensory evidence (the degree of temporal alignment), rather than the prior of a common event as we did in the present study.

An fMRI study^19^ reported that presenting known audio-visual stimulus combinations with a stimulus onset asynchrony generated an enhanced activation in the auditory cortex when vision was leading and an enhanced activation in the visual cortex when the sound was leading compared to a synchronous presentation. The authors interpreted this activation as a prediction error that would not be expected if the audio-visual combination is unknown. Such a model, however, would rather predict better simultaneity judgments for known than for unknown crossmodal stimuli: known stimuli would produce an error signal whereas unknown crossmodal stimuli would not. However, we and others^11, 16^ found the opposite. It might be speculated that what Lee and Noppeney^19^ observed was a predictive activation of the sensory areas associated with the lagging stimulus partly due to the previously learned crossmodal statistics. Such priming effects, which have been recently found in a study with infants using an omission paradigm^20^, would result in a temporally overlapping activation of auditory and visual areas for asynchronous stimuli that are highly associated, which might increase the likelihood of perceived simultaneity. In contrast, non-associated or unknown asynchronous cross-modal events would not generate a predictive activation, resulting in temporally more distinct brain activation patterns, which in turn could lead to better simultaneity judgment performance for non-associated or unknown crossmodal stimuli compared to associated or known crossmodal stimuli. This prediction could be tested by extending the fMRI study of Lee and Noppeney^19^ by using both known and unknown audiovisual combinations and assessing brain activation in parallel to SJ thresholds.

An additional finding of the present study was that newly combined audio-visual stimuli were more likely perceived as simultaneous than new auditory and new visual stimuli that were not previously encountered. Note that for the newly combined videos the movement dynamics were predictable by both the auditory and visual element, while movement dynamics were unpredictable for the new crossmodal stimuli. Thus, this result might suggest that priors integrate crossmodal co-occurrence probabilities for multiple stimulus dimensions of a crossmodal event rather than for a unified event.

Finally, we would like to point out that our results are distinct from those typically found in studies on crossmodal temporal discrimination training^21^ or on temporal cross-modal recalibration^22^. In the latter, participants are typically exposed to crossmodal stimuli with a constant temporal asynchrony. Compared to a pre-adaptation assessment, participants typically shift their simultaneity judgments towards the asynchrony experienced during the adaptation phase. In contrast, in the present study auditory and visual stimuli were always presented in synchrony during the learning phase. Thus, the prior of an auditory and visual stimulus to co-occur was manipulated rather than the temporal alignment. In crossmodal temporal discrimination training studies, typically only *one* crossmodal stimulus is used while the stimulus onset asynchrony is manipulated and participants receive feedback about the correctness of their response during training.

In sum, our data suggest that learning statistics about the co-occurrence of sensory stimuli results in a change in the prior that two sensory inputs were caused by the same event. As a result, the ability to separate these events decreases, despite unchanged sensory evidence.

## Methods

### Participants

Fourteen healthy participants (eight male and six female, aged from 19–28 years, mean age 21 years) took part. All participants were students of the University of Hamburg. None of them reported a history of neurological disease. All participants had normal hearing and normal or corrected-to-normal vision. Participants gave written informed consent and were compensated for participation by means of money or course credit. The study was approved by the ethical board of the German Psychological Society (DGPs, Nr. 102009). The experimental procedures were performed in accordance with the guidelines of the ethics committee.

### Stimuli

Stimuli were videos of moving artificial figures combined with a sound. Eighteen different artificial figures (see supplementary Figure S1) were constructed with Adobe Illustrator®. Each figure consisted of two overlapping shapes which were either vertically (5 × 7.5 cm) or horizontally (7.5 × 5 cm) aligned and which used one of three color combinations (1: pink, yellow, brown, green; 2: light blue, grey, orange, red; 3: dark-blue, light-green, purple, dark-orange). One of four facial expressions (sad, friendly, neutral or surprised) was superimposed on top of the shapes. All figures were presented on a black background and had the same starting position at the center of the screen. Figures moved similar distances in one of four different directions (to the right, to the left, upwards or downwards) and one of four different ways (continuous: one smooth movement; two steps: figure jumps two steps; one step: figure jumps in one step; or vibrating: small movements to the left and to the right). All features were randomly assigned to all stimuli and all conditions (see Supplementary Table S1 for an overview of all stimulus conditions).

Eighteen sound files were selected from a published database^17^. The sounds selected for the present study were chosen to match the movement types of the visual stimuli (for example, a figure displaying a continuous movement was matched to a sound file displaying a continuous sound; a jumping sound was matched to a jumping visual stimulus etc.). The durations of the sound files were adjusted (using the software Goldwave®) to create on- and offset synchrony to the movement displayed in the visual stimuli. Auditory stimuli were saved with a sampling rate of 22 kHz (16-bit, mono) in WAV-format. All auditory sound files were normalized to an equal dB-level.

Thirty audio-visual videos were created with Adobe Premiere® (see Supplementary Table S1 for an overview of all conditions and stimuli). Each video had a duration of 1000 ms: 400 ms of movement in a certain direction, 200 ms of the figure standing still, followed by 400 ms of movement back to the starting position.

### Design

There were five conditions (see Supplementary Table S1): The first condition, *audio-visual learned (L)*, contained six videos (A1V1-A6V6; see Supplementary Figure S1 and Supplementary Table S1). The second condition, *audio-visual newly combined (NC)*, contained the same audio- and visual files as the learned condition (A1-A6 and V1-V6), but combined into different audio-visual combinations (e.g., A1V6, A2V5 etc). Note, that we took care that the auditory and visual dynamics matched in the newly combined videos as in the learned videos. The third condition, *audio-visual visual_learned (V-l)*, contained the learned visual material V1-V6 combined with new sounds A13-A18. The fourth condition, *audio-visual auditory_learned (A-l)*, used the learned auditory material A1-A6 combined with new visual stimuli V13-V18. The last condition, *audio-visual new (N)*, contained completely new auditory and visual material: A7V7-A12V12.

For each of these thirty audio-visual combinations, nine SOAs were created, in steps of 100 ms: −400, −300, −200, −100, 0, 100, 200, 300 and 400 ms. The negative SOAs, −400 to −100, refer to auditory stimulus onset preceding the visual stimulus onset, and the positive SOAs refer to visual stimulus onset preceding the auditory stimulus onset. The 0 ms SOA denotes synchronous presentation of auditory and visual information.

### Procedure

Stimuli were presented with Presentation version 16.3. (Neurobehavioral Systems, Inc., Berkeley, CA). Participants sat 85 cm from the computer screen. They were informed about the experiment and task prior to participation. The experimental session contained three phases: during the first phase, the learning phase, participants saw only the six audio-visual videos from the learned (L) condition (A1V1-A6V6). Each video was presented three times in a row, and each set of all six videos was presented three times as well, so that each video was seen nine times by each participant. Presentation of the videos was self-paced as participants started the presentation of each video with a button press. Participants were instructed to pay attention to the audio-visual combination of each video. All videos during the learning phase were presented with an SOA of 0 ms (i.e., auditory and visual stimuli were always presented synchronously).

During the second phase, the actual testing phase, participants saw two blocks of 270 videos each (5 conditions x 6 videos per condition x 9 SOAs; see Design). Each video was preceded by a 500 ms presentation of a smiley at the center of the screen. This was to make sure that participants focused their attention on the computer screen prior to the onset of the stimuli. Presentation of the leading stimulus component started immediately after smiley-offset. The task of the participant was to decide whether or not the auditory and visual information was presented at the same time (simultaneity judgment task). They answered yes or no by pushing the left and right mouse button (buttons were counterbalanced over participants). They were told to respond only after the video was finished. Following the response, there was a variable inter-trial interval (ITI) between 1000 and 3000 ms (equal distribution) before smiley-onset of the next trial. Each of the two blocks lasted approximately 15 min.

During the last phase of the experiment, we tested participants’ memory of the six videos from the learned (L) condition that were presented in the first phase. Participants saw all thirty videos (with synchronous audio-visual presentation) once and indicated which of the videos they had learned at the beginning of the experimental session. Only participants who correctly recognized all six learned videos and had less than two false alarms on the 24 non-learned videos were taken into the final analysis. All three phases of the experiment were conducted in a sequential manner, with only short breaks in between. Total testing time for a complete experimental session was approximately one hour.

### Data analysis

Reaction times and perceived simultaneity judgments were collected. Perceived simultaneity judgments were calculated by taking the proportion of trials for each condition and each SOA in which a participant reported a simultaneous presentation of the audio-visual stimuli. Separate ANOVAs for the proportions of perceived simultaneity and reaction times were calculated which comprised the within-participant factors First Modality (2 levels: auditory, visual), SOA (4 levels: 100, 200, 300, 400 ms) and Condition (5 levels: audio-visual learned (L), audio-visual newly combined (NC), audio-visual visual_learned (V-l), audio-visual auditory_learned (A-l), and audio-visual new (N)). In addition, temporal binding windows (TBWs) were derived for each participant and condition from the proportions of perceived simultaneity at each SOA. For this purpose, data from the auditory-leading (−400 to 0 ms) and from the visual-leading (0 to 400 ms) SOAs were separately fitted by logistic regressions. Data points were then split at the SOA at which these two sigmoid functions crossed and refitted with two new sigmoids; this process was iterated until the two fittings converged^16, 23^. TBWs were estimated from the final fittings as the difference (in ms SOA) between the right and the left curve at 75% perceived simultaneity. Individual TBWs were then submitted to a one-way repeated-measures ANOVA to test for significant differences between conditions.

In all analyses, significant interactions or main effects were followed up by two-tailed dependent *t*-tests (pairwise comparisons). An alpha level of 0.05 was used for all statistical tests. Huynh-Feldt correction for violations of the sphericity assumption was applied and the corrected probabilities are reported where appropriate^24^. Five videos per condition were randomly chosen for the analyses, due to an error that was detected in one of the video files. This made sure that each condition was represented by the same number of videos for the analyses.

## Electronic supplementary material

Supplementary information

## References

[1] Stein BE, Stanford TR (2008). Multisensory integration: current issues from the perspective of the single neuron. Nat. Rev. Neurosci..

[2] Miller J (1982). Divided attention: evidence for coactivation with redundant signals. Cogn. Psychol.

[3] Stein, B. E. The new handbook of multisensory processing (MIT Press, 2012).

[4] Welch RB, Warren DH (1980). Immediate perceptual response to intersensory discrepancy. Psychol. Bull..

[5] Föcker J, Hölig C, Best A, Röder B (2011). Crossmodal interaction of facial and vocal person identity information: an event-related potential study. Brain Res..

[6] Shams, L. Early integration and Bayesian causal inference in multisensory perception in *The Neural Bases of* Multisensory Processes (eds Murray, M. M. & Wallace, M. T.) 217-231 (CRC Press/Taylor & Francis, 2012). 22593872

[7] Ten Oever S (2016). The COGs (context, object, and goals) in multisensory processing. Exp. Brain Res..

[8] Vatakis A, Spence C (2007). Crossmodal binding: evaluating the “unity assumption” using audiovisual speech stimuli. Percept. Psychophys..

[9] Van Wassenhove V, Grant KW, Poeppel D (2007). Temporal window of integration in auditory-visual speech perception. Neuropsychologia.

[10] Ten Oever S, Sack AT, Wheat KL, Bien N, van Atteveldt N (2013). Audio-visual onset differences are used to determine syllable identity for ambiguous audio-visual stimulus pairs. Front. Psychol..

[11] Vatakis A, Ghazanfar AA, Spence C (2008). Facilitation of multisensory integration by the “unity effect” reveals that speech is special. J. Vis..

[12] Vatakis A, Spence C (2008). Evaluating the influence of the ‘unity assumption’ on the temporal perception of realistic audiovisual stimuli. Acta. Psychol. (Amst.).

[13] Arrighi R, Alais D, Burr D (2006). Perceptual synchrony of audiovisual streams for natural and artificial motion sequences. J. Vis..

[14] Giard MH, Peronnet F (1999). Auditory-visual integration during multimodal object recognition in humans: a behavioral and electrophysiological study. J. Cogn. Neurosci.

[15] Kording KP (2007). Causal inference in multisensory perception. PLoS One.

[16] Stevenson RA, Wallace MT (2013). Multisensory temporal integration: task and stimulus dependencies. Exp. Brain Res..

[17] Schneider TR, Engel AK, Debener S (2008). Multisensory identification of natural objects in a two-way crossmodal priming paradigm. Exp. Psychol..

[18] Wallace MT (2004). Unifying multisensory signals across time and space. Exp. Brain Res..

[19] Lee H, Noppeney U (2014). Temporal prediction errors in visual and auditory cortices. Curr. Biol..

[20] Emberson LL, Richards JE, Aslin RN (2015). Top-down modulation in the infant brain: learning-induced expectations rapidly affect the sensory cortex at 6 months. Proc. Natl. Acad. Sci. USA.

[21] Powers AR, Hillock AR, Wallace MT (2009). Perceptual training narrows the temporal window of multisensory binding. J. Neurosci..

[22] Chen L, Vroomen J (2013). Intersensory binding across space and time: a tutorial review. Atten. Percept. Psychophys..

[23] Stevenson RA (2014). Multisensory temporal integration in autism spectrum disorders. J. Neurosci..

[24] Huynh H, Feldt LS (1970). Conditions under which mean square ratios repeated measurements designs have exact F-distributions. J. Am. Stat. Assoc.

