# Supplementary material for: Experience with crossmodal statistics reduces the sensitivity for audio-visual temporal asynchrony
Source: Sci Rep. 2017 May 3;7:1486. doi: 10.1038/s41598-017-01252-y (PMC5431144; doi:10.1038/s41598-017-01252-y)
Supplement: Supplementary file 1 — Supplementary information [file 41598_2017_1252_MOESM1_ESM.pdf]

Supplementary Information

"Experience with crossmodal statistics reduces the sensitivity for audio-visual temporal asynchrony"

Boukje Habets, Patrick Bruns & Brigitte Röder

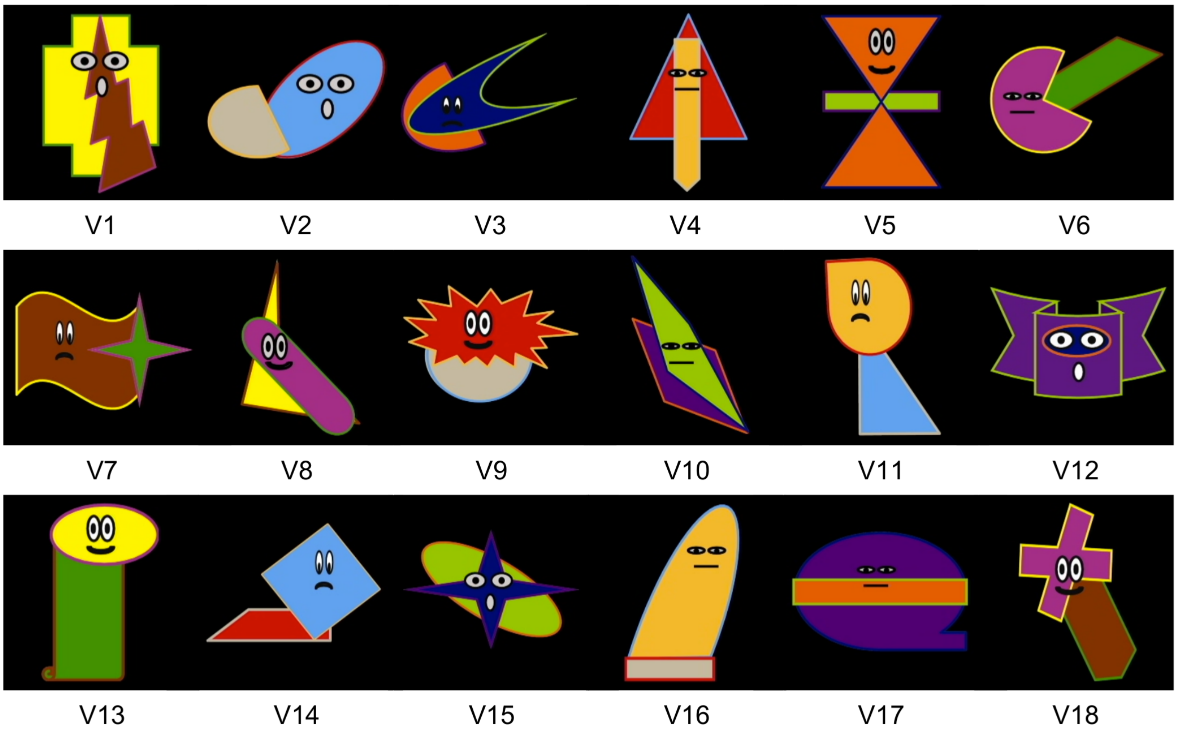

Figure S1. Visual stimuli (see also Supplementary Table S1).

Table S1. Conditions and stimuli.

Condition	Stimulus	Alignment	Color	Face	Direction	Way	Audio
**L**	A1V1	Vertical	1	Surprised	Left	Continuous	“video”
	A2V2	Horizontal	2	Surprised	Left	2 steps	“musicbox”
	A3V3	Horizontal	3	Sad	Right	1 step	“icecubes”
	A4V4	Vertical	2	Neutral	Down	1 step	“cork”
	A5V5	Vertical	3	Smiling	Up	2 steps	“motorsaw”
	A6V6	Horizontal	1	Neutral	Down	Vibrating	“teeth”
**NC**	A6V1	Vertical	1	Surprised	Left	Continuous	“teeth”
	A5V2	Horizontal	2	Surprised	Left	2 steps	“motorsaw”
	A4V3	Horizontal	3	Sad	Right	1 step	“cork”
	A3V4	Vertical	2	Neutral	Down	1 step	“icecubes”
	A2V5	Vertical	3	Smiling	Up	2 steps	“musicbox”
	A1V6	Horizontal	1	Neutral	Down	Vibrating	“video”
**V_l**	A13V1	Vertical	1	Surprised	Left	Continuous	“tone”
	A14V2	Horizontal	2	Surprised	Left	2 steps	“handsaw”
	A15V3	Horizontal	3	Sad	Right	1 step	“rooster”
	A16V4	Vertical	2	Neutral	Down	1 step	“trumpet”
	A17V5	Vertical	3	Smiling	Up	2 steps	“cuckoo”
	A18V6	Horizontal	1	Neutral	Down	Vibrating	“water”
**A_l**	A1V13	Vertical	1	Smiling	Left	Continuous	“video”
	A2V14	Horizontal	2	Sad	Left	2 steps	“musicbox”
	A3V15	Horizontal	3	Surprised	Right	1 step	“icecubes”
	A4V16	Vertical	2	Neutral	Down	1 step	“cork”
	A5V17	Horizontal	3	Neutral	Up	2 steps	“motorsaw”
	A6V18	Vertical	1	Smiling	Down	Vibrating	“teeth”
**N**	A7V7	Horizontal	1	Sad	Right	2 steps	“morse”
	A8V8	Vertical	1	Smiling	Up	1 step	“computer”
	A9V9	Horizontal	2	Smiling	Up	Continuous	“flute”
	A10V10	Vertical	3	Neutral	Down	Continuous	“bagpipe”
	A11V11	Vertical	2	Sad	Right	Vibrating	“car”
	A12V12	Horizontal	3	Surprised	Left	Vibrating	“violin”

Note. L = audio-visual learned; NC = audio-visual newly combined; V-l = audio-visual visual_learned; A-l = audio-visual auditory_learned; N = audio-visual new.

Table S2. Mean proportion of simultaneity judgments per condition and SOA (with SEMs).

SOA	L	NC	V-l	A-l	N
-400	0.09 (0.04)	0.10 (0.03)	0.08 (0.05)	0.09 (0.05)	0.04 (0.03)
-300	0.27 (0.08)	0.18 (0.05)	0.17 (0.07)	0.21 (0.05)	0.16 (0.06)
-200	0.45 (0.09)	0.53 (0.08)	0.37 (0.09)	0.47 (0.09)	0.34 (0.06)
-100	0.83 (0.06)	0.75 (0.06)	0.65 (0.08)	0.63 (0.09)	0.58 (0.07)
0	0.91 (0.03)	0.87 (0.04)	0.83 (0.04)	0.80 (0.05)	0.80 (0.05)
100	0.98 (0.02)	0.91 (0.04)	0.91 (0.04)	0.86 (0.04)	0.81 (0.07)
200	0.93 (0.05)	0.88 (0.04)	0.88 (0.04)	0.86 (0.03)	0.88 (0.04)
300	0.85 (0.06)	0.71 (0.07)	0.69 (0.08)	0.67 (0.07)	0.64 (0.07)
400	0.38 (0.09)	0.31 (0.09)	0.36 (0.08)	0.27 (0.08)	0.28 (0.08)

Note. SOA = stimulus onset asynchrony (negative values = auditory stimulus first, positive values = visual stimulus first); L = audio-visual learned; NC = audio-visual newly combined; V-l = audio-visual visual_learned; A-l = audio-visual auditory_learned; N = audio-visual new.
